# Left ventricular torsional dynamics post exercise for LV diastolic function assessment

**DOI:** 10.1186/1476-7120-12-8

**Published:** 2014-02-15

**Authors:** Muhammad Asrar ul Haq, Vivek Mutha, Tina Lin, Konstantinos Profitis, Zoe Tuer, Kwang Lim, David L Hare, Chiew Wong

**Affiliations:** 1 Department of Cardiology, The Northern Hospital, 185 Cooper Street, Melbourne, Victoria 3076, Australia; 2 Austin Health, Melbourne, Australia; 3 Department of Medicine, University of Melbourne, Melbourne, Australia

**Keywords:** Diastolic dysfunction, HFPEF, Torsion, Speckle tracking

## Abstract

**Aims:**

2D speckle tracking echocardiography allows for assessment of left ventricular (LV) torsional deformation as a composite function of the radial, longitudinal and circumferential fibres. We test the hypothesis that post-exercise LV torsional dynamics are more sensitive markers for myocardial dysfunction than resting measures, and better predictors for exercise capacity compared to post-exercise LV diastolic filling pressure (E/e’).

**Methods:**

We studied 88 patients referred for stress echocardiogram. Treadmill exercise was performed using Bruce protocol, and echo images were acquired using GE Vivid 7. LV rotational dynamics were analysed by speckle tracking method using the GE ECHOPAC software. Tertiles were defined according to exercise capacity measured by the achieved metabolic equivalents (METS) adjusted for age and gender. Comparison was made between LV torsional dynamics and E/e’ to correlate with METS to predict exercise capacity.

**Results:**

Mean age of the study population was 58 years, 48% females. Patients with systolic dysfunction or evidence of ischaemia were excluded from the analysis. No significant correlation was found between METS and LV torsion measures at rest. There was statistically significant correlation between METS and post-exercise LV torsion (r=0.34, p=0.001), twist velocity increase (r=0.27, p=0.01), and incremental change in torsion (r=0.22, p<0.05). In addition, a correlation was also shown between post-exercise E/e’ and METS (r=-0.33, p=0.002).

**Conclusion:**

Post-exercise LV torsional dynamics correlate with exercise capacity and may be a useful tool for assessing LV myocardial function in subjects with normal LVEF.

## Background

Diagnosing and monitoring of left ventricular (LV) dysfunction can be a challenge at early stages of the myocardial disease before a reduction in the LV contractility measured using the conventional measures or blood pool information for ejection faction (EF). 2D speckle tracking is a newer technique that enables quantification of function of myocardial fibres at the local level taking account the rotational motion of the myocardium in different directions. It has been validated non-invasively against magnetic resonance imaging (MRI) tagging and invasively using sonomicrometry [1-4].

Stress Echocardiography using the newer techniques to assess LV tissue velocities, strain and torsion has been shown to be more sensitive in predicting LV diastolic dysfunction [5]. LV diastolic filling pressure (E/e’) estimated with Doppler echocardiography becomes elevated during exercise. It has been well validated with cardiac catheterisation findings and been shown to predict exercise capacity [6]. During the systole phase of cardiac cycle, the base rotates in a clockwise direction when viewed from the apex, whilst the apex rotates counter clockwise resulting in the twist motion of the LV. Rapid untwisting during diastole causes a suction effect and contributes to the ventricular filling. This phenomenon has been described in both animals and humans [7,8]. The rapid untwisting of left ventricle becomes particularly important during the exercise activity when due to rapid heart rate, a more efficient LV filling is required and failure to increase LV torsion may attenuate LV diastolic performance, leading to elevation of LV filling pressures. We examined LV torsional dynamics pre- and post-exercise and compared to post-exercise E/e’ to test the hypothesis that post-exercise LV torsional dynamics are more sensitive markers for myocardial dysfunction than resting measures, and better predictors for exercise capacity and myocardial dysfunction compared to post-exercise LV diastolic filling pressure (E/e’).

## Methods

### Study population

Successive patients over 18 year of age who were referred for exercise stress echocardiography to investigate for symptoms of dyspnoea or chest pain were evaluated if they completed the test. Exclusion criteria included a prior history of myocardial infarction or known coronary artery disease, ischaemic ECG changes or anginal symptoms during the test, induced or fixed wall motion abnormality on echocardiogram, systolic dysfunction defined as ejection fraction (EF) of less than 50%, significant valvular heart disease, conditions requiring electric pacemaker or defibrillator, atrial fibrillation (AF), inability to walk on treadmill, or history of significant respiratory disease. Patients with suboptimal echo images defined as inadequate frame rate, poor tracking at high heart rate, or inability to visualise all 17 segments were also excluded. Significant valvular disease was defined as any valvular disease of more than mild severity, and significant respiratory disease based on previous clinical diagnosis, history of inhalers use, smoking history of more than 10 pack years, or suggestive clinical examination.

### Exercise stress test

Symptom-limiting (maximum fatigue or dyspnoea) treadmill exercise tests were performed using the Bruce protocol. Exercise capacity was estimated in metabolic equivalents (METS) [9,10], based on the speed and grade of the treadmill on a modified Bruce protocol [11]. Patients were divided into three groups according to the percentage predicted exercise capacity adjusted for age and gender [9,10] based on the tertiles of achieved metabolic equivalents, METS (≤100%, 101-125%, ≥126%).

### Measurements

**Figure 1:**
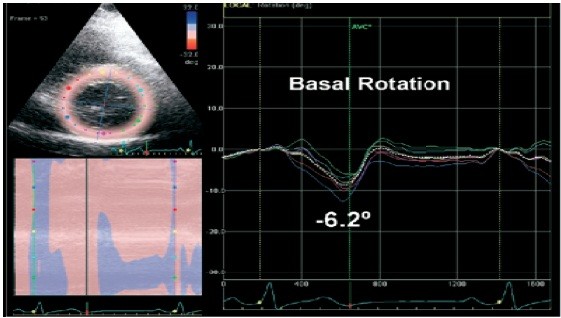
Basal and apical rotation curves showing the peak twist degrees.

Diastolic and systolic parameters at rest and post exercise were assessed. Echocardiography images were acquired immediately post-exercise in supine posture using GE Vivid 7 set at high frame-rate of >100 frames/second, and an angle of ≤15° for apical views. GE ECHOPAC software was used for offline analysis. The LVEF was measured with the modified biplane Simpson’s method [12]. E/e’ ratio was calculated for estimated filling pressure [13] by measuring the early transmitral inflow (E) and early velocity of mitral valve septal annulus (e’) by pulsed wave tissue Doppler method [14]. Parasternal short axis images were analysed at basal (mitral valve level) and apical levels (furthest possible view distal to the papillary muscle level) [15] of the LV using 2D speckle tracking echocardiography by two independent operators in a blinded fashion. Utmost care was taken to acquire the basal and apical images using different planes and not merely changing the angle at the same acquisition point. Speckles in 2D images as tissue markers were used to track LV contraction at rest and post exercise [2,4,5]. More than 20 markers were selected along the endocardium and adjusted manually where appropriate. Thickness of the region of interest (ROI) was adjusted to include the myocardium. Using recommended definitions [16,17], LV twist was calculated as difference between clockwise rotation of the base and counter-clockwise rotation of the apex (Figure 1) expressed in degrees (°), while torsion was defined as LV twist per unit length and was obtained by dividing the total twist degree by the length of the ventricle (°/cm) [18]. LV Length was defined as the maximum distance between the midpoint of the mitral annulus to the apex in the apical two- and four-chamber views [12].

### Statistics

Correlation of LV parameters include the LV torsional measures, LV diastolic measure, and filling pressure to achieved METS was determined. Paired t-test was used to compare normal distribution data and the linear regression (Pearson’s coefficient) was performed to test correlations between echocardiographic findings and the clinical features. One way ANOVA and Pearson Chi-Square tests were used to compare characteristics between different groups. Two tailed values of P<0.05 were considered statistically significant.

## Results

Out of 249 patients screened, 161 were excluded (positive stress test: 53; systolic dysfunction: 44; history of ischaemic heart disease: 37; valvular disease: 11; significant respiratory disease: 7; miscellaneous: 9). Clinical characteristics of the total 88 analysed patients are summarised in Table 1. Mean age of the patients was 58±12 years, and 48% were women. Average body mass index (BMI) of the cohort was 29±5.8. Average METS achieved were 8.9 with 83% of people reaching at least 85% of predicted exercise capacity. The resting EF was 63±6%, with an average LV mass of 127±41 and length 7.6±0.89. Pre-exercise E/e’ was found to be 9.6±3.8 which increased to 12.1±4 post exercise (p=0.02). BMI was correlated with LV mass (r=0.42, p=0.01).

**Table 1:** Clinical characteristics of the studied population categorised into 3 subgroups, shown as mean ± SD

**Group**	**All patients (n = 88)**	**1 (n = 28)**	**2 (n = 30)**	**3 (n = 30)**	**p value**
		**Predicted exercise capacity**	**Predicted exercise capacity**	**Predicted exercise capacity**	
		**≤100%**	**101-125%**	**≥126%**	
Age (yrs)	60 ± 12	58 ± 13	62 ± 11	60 ± 11	p=0.4
Males/Females	46/42	14/14	14/16	18/12	p=0.6
History of hypertension	39	18	14	7	p<0.01
History of diabetes mellitus	23	11	5	7	p<0.01
Weight (kg)	78 ± 17	81 ± 18	81 ± 21	74 ± 13	p=0.5
Height (m)	1.65 ± 0.1	1.63 ± 0.1	1.63 ± 0.1	1.6 ± 0.1	p=0.8
BMI (kg/m^2^)	29 ± 5.8	29 ± 6.2	32 ± 6.6	26 ± 2.6	p=0.1
METS achieved	8.9 ± 2.9	6.2 ± 2.4	9.3 ± 2	11.3 ± 1.8	p<0.000

The apical rotation and apical rotational velocity were significantly higher post exercise (4.7±3.6 vs. 3.8±2.7°, p=0.02; and 66±37 vs. 46±27, p<0.001 respectively). However the basal rotation did not change post exercise. When comparison made across the 3 groups based on their achieved workload adjusted for age and gender (Table 2), LV torsion after exercise was different between the three groups increasing with higher exercise capacity (1 ±0.5, 1.4 ±0.6, 1.5 ±0.6, p=0.006). The increment between the pre and post exercise apical rotation was not significant in people with lower exercise capacity (group 1) whereas the group 2 & 3 patients who achieved more than 100% of their predicted METS had significantly increased apical rotation (5.1±3 vs. 3.2±2 and 5.8±4 vs. 3.8±3, p<0.05). The twist velocity change with exercise increased across the groups and were statistically significant, in subgroup 2 (p=0.007) and subgroup 3 (p=0.002).

**Table 2:** Left ventricular (LV) rotation and torsion characteristics of subgroups according to percentage predicted exercise capacity

**Tertiles**	**1 (n = 28)**	**2 (n = 30)**	**3 (n = 30)**	**ANOVA**
	**Predicted exercise capacity**	**Predicted exercise capacity**	**Predicted exercise capacity**	
	**≤100%**	**101-125%**	**≥126%**	
LV Torsion at Rest (°/cm)	1.2 ± 0.6	1.2 ± 0.5	1.3 ± 0.5	NS
LV Torsion post exercise (°/cm)	1.0 ± 0.5	1.4 ± 0.6	1.5 ± 0.6	p=0.006
LV Torsion change (Δ)	-0.13 ± 0.7	0.16 ± 0.6	0.17 ± 0.6	NS
LV apex rotation at Rest (°)	3.9 ± 3.0	3.2 ± 1.8	3.8 ± 3.1	NS
LV apex rotation post exercise (°)	3.5 ± 2.7	5.1 ± 3.1	5.8 ± 4.2	p=0.04
LV apex rotation change (Δ)	-0.4 ± 4.0	1.6 ± 3.4	1.8 ± 3.4	p=0.04
LV apex rotation velocity at Rest (°/s)	48 ± 28	38 ± 22	52 ± 30	NS
LV apex rotation velocity post exercise (°/s)	53 ± 32	75 ± 37	69 ± 39	NS
LV apex rotation velocity change (Δ)	5 ± 39	35 ± 44	15 ± 48	NS
LV base rotation at Rest (°)	5.0 ± 2.4	5.3 ± 3.3	6.3 ± 2.9	NS
LV base rotation post exercise (°)	4.5 ± 2.7	4.9 ± 2.7	5.9 ± 3	NS
LV base rotation change (Δ)	-0.6 ± 3.3	-0.5 ± 3.7	-0.5 ± 4.3	NS
LV base rotation velocity at Rest (°/s)	69 ± 36	42 ± 28	55 ± 28	NS
LV base rotation velocity post exercise (°/s)	61 ± 30	65 ± 30	71 ± 34	NS
LV base rotation velocity change (Δ)	-8.3 ± 40	25 ± 33	14 ± 47	NS

The change in post exercise E/e’ (14.2±5.2, 12.8±4.8, 11.1±3, p<0.05) was statistically significant between the three groups, while the baseline E/e’ between the three groups was not found significant (9.5±2.8, 9±3.3, 10.1±5.2, p=0.8).

There was correlation of body mass index (BMI) with post-exercise LV torsion (r=-0.31, p<0.005), Increment (∆) in LV torsion (r=-0.34, p=0.001), post exercise basal rotation (r=-0.25, p=0.017), basal rotation ∆ (r=-0.27, p=0.011) and LV twist velocity change ∆ (r=-0.52, p<0.0001). The LV torsion ∆ was also negatively correlated with age (r=-0.23, p<0.05). No significant correlation between METS and LV torsion at rest was found. There was a statistically significant correlation demonstrated between LV torsion post-exercise and METS (r=0.34, p=0.002). Post-exercise E/e’ also showed correlation with METS (r=-0.33, p=0.002). A weaker but statistically significant correlation was seen between METS and rotation velocity incremental (r=0.27, p=0.01), and torsion incremental (r=0.22, p<0.05).

## Discussion

In our study, we examined the LV rotational dynamics, LVEF, and estimated filling pressures in a cohort of patients who were referred for exercise stress echocardiogram, using the newer advanced echocardiographic quantification. The newer LV measures with Tissue Doppler Imaging and 2D speckle tracking has improved our understanding of the myocardial mechanics and hence more accurate assessment of LV function. These have enabled us to identify the early changes in the myocardial mechanics which translates into early detection of disease process and might explain symptoms of exertional dyspnoea or reduced exercise tolerance [2,5,19]. Using these technologies we have demonstrated a failure to increase the apical twist and rotational velocities post exercise as a quantifiable echocardiographic phenomenon present in patients with reduced exercise tolerance and comparable with the validated non invasive LV filling pressure estimates obtained with echocardiogram. This may suggest that the abnormalities of LV torsion and twist velocities occur before the reduction in the LVEF, a blood pool information, although further studies would be required to confirm this and to assess its prognostic and clinical significance.

Earlier LV torsion dynamics studies suggested that this complex phenomenon is directly related to myocardial muscle fibre orientation [20,21]. The multi-layered fibre arrangements in longitudinal, circumferential and spiral directions interact to produce a twisting and untwisting motion when myocardium contracts. This untwisting motion during early diastole causes a negative intraventricular pressure leading to rapid LV filling [5,7]. Rapid untwisting of left ventricle becomes particularly important during exercise when the increased heart rate demands a more efficient LV filling [5].

While resting LVEF did not predict exercise tolerance in our cohort with no systolic dysfunction or prior diagnosis of heart failure, our study demonstrated that post exercise LV torsional dynamics may predict exercise capacity. Alteration in LV torsion in response to changes in preload and afterload has been postulated [15,22]. These haemodynamic consequences of exercise may be the driving force for the changes in the LV torsion, which became apparent with exercise challenge, indicating an insensitivity of resting LV torsional parameters. Patients who had better exercise capacity could augment LV apical rotation as well as LV twist velocity post exercise more than those with lower exercise capacity. As previously demonstrated by Notomi et al. [5], we have shown that LV twist velocity increases on exercise. Similar to previous findings [23,24], our findings suggest that the increment in LV torsion post exercise is mainly contributed by apical rather than basal rotation, and failure of an increase post exercise could lead to elevated LV filling pressure. Reduced LV apical rotation and slower LV twist may explain the delayed early diastolic filling secondary to reduced negative intraventricular pressure suggesting the impaired relaxation and elevated filling pressure in patients with HFPEF may be a consequence of abnormal torsional dynamics. LV apical rotation post exercise or during acute presentation can therefore be one of the major determinants and a sensitive marker for diagnosing HFPEF.

Furthermore, in our study, age was inversely proportional to LV torsion whereas BMI, which was correlated with LV mass, was negatively associated with LV torsion increment. Impairment in diastolic parameters with increasing age is well recognised and in fact higher values have been recommended as normal range in older age groups, e.g. for deceleration time (DT) as a marker of impaired relaxation [25,26]. Age related reduction in LV torsion may explain the changes in other established parameters to measure diastolic function. Similarly, BMI has previously been associated with reduced diastolic function as evidenced by myocardial tissue velocity e’ and strain patterns, independent of EF [27]. This again may be a consequence of LV torsion impairment related to subclinical changes in LV structure with increasing BMI, leading to failure of LV torsion increment with exercise causing exertional symptoms.

While our study attempted to make measurements in the face of hemodynamic perturbation, there are few limitations to consider. First of all it is a cross sectional analysis involving a clinical population referred for stress echo to assess common symptoms including dyspnoea and chest pain, however a further trial on patients presenting with exertional dyspnoea involving formal cardiopulmonary testing should be conducted to account for the other variables to predict exercise capacity in a controlled environment. This is particularly important given a rather weak correlation despite statistical significance in our study. Secondly, as it is known that hypertension and diabetes affect LV twist, a study powered for subgroup analysis could be useful to see the impact on the results. Similarly, adjusting rotational mechanics for the peak systolic blood pressure may be desirable owing to its potential effect on twist. Thirdly, estimated METS are relatively crude parameter for assessing peak exercise capacity. Future studies incorporating more accurate measures e.g. VO2-peak assessments are required to validate the results further.

## Conclusion

Myocardial mechanics such as LV torsion on exercise provocation is one of the important determinants of exertional dyspnoea, and may add valuable information in assessing cardiac contribution to exercise capacity. Measurement of LV torsion dynamics post-exercise is superior to the resting measures, and can be a useful tool in the assessment for myocardial dysfunction beyond the LV filling measure in patients with HFPEF. Further large-scale studies are required to validate the findings.

## Competing interests

The authors declare that they have no competing interests.

## Authors’ contributions

All authors contributed equally. All authors read and approved the final manuscript.
